# Functional analysis of MKP-1 and MKP-2 in breast cancer tamoxifen sensitivity

**DOI:** 10.18632/oncotarget.1795

**Published:** 2014-03-13

**Authors:** Kelly K. Haagenson, Jessica Wei Zhang, Zhengfan Xu, Malathy P.V. Shekhar, Gen Sheng Wu

**Affiliations:** ^1^ Department of Oncology, Wayne State University School of Medicine, Detroit, MI; ^2^ Barbara Ann Karmanos Cancer Institute, Detroit, MI; ^3^ Department of Pathology, Wayne State University School of Medicine, Detroit, MI

**Keywords:** MKP-1, MKP-2, ERK, tamoxifen sensitivity, breast cancer

## Abstract

Increased activation of ERK signaling has been reported in breast cancer models of acquired tamoxifen resistance. Here, we examined the expression of Mitogen-Activated Protein Kinase Phosphatases (MKPs) 1 and 2 following tamoxifen treatment and the effects of MKP-1/MKP-2 overexpression on tamoxifen sensitivity. Treatment of MCF7 breast cancer cells with tamoxifen increased MKP-2, but not MKP-1, protein levels. Overexpression of MKP-1 or MKP-2 inhibited estrogen-induced MCF7 cell proliferation compared to vector controls. MCF7-MKP-2 cells displayed significantly increased sensitivity to tamoxifen as compared to vector control or MCF7-MKP-1 cells. MKP-1 or MKP-2 overexpression eliminated ERK1/2 phosphorylation, suggesting that decreases in estrogen-induced proliferation of MKP-1 and MKP-2 overexpressing cells are due to ERK1/2 dephosphorylation. JNK1/2 activation was not detectable in any of these cells. These data suggest that tamoxifen-induced death of these cells is not dependent upon JNK signaling, but rather that ERK is the major MAPK driving their proliferation. MCF7-TAMR cells express higher levels of MKP-2 mRNA and protein than MCF7 cells. MKP-2 and phospho-ERK1/2 proteins are constitutively expressed in MCF7-TAMR cells, and activated JNK1/2 is not detectable. These data suggest that MKP-2 rather than MKP-1 is tamoxifen-regulated and that the elevated expression of MKP-2 in MCF7-TAMR cells potentially functions to restore tamoxifen sensitivity.

## INTRODUCTION

Approximately 70% of breast tumors express estrogen receptor alpha (ERalpha) [1, 2]. For women who present with ERalpha-positive tumors, first-line therapy involves treatment with tamoxifen. Tamoxifen mimics the binding of estrogen to the estrogen receptor and inhibits its function. When used in an adjuvant setting, tamoxifen reduces the chance of developing recurrent disease by 40-50% [3]. Approximately 30-50% of women with metastatic disease will experience temporary remission while being treated with tamoxifen [3, 4]. Unfortunately, most of these women will eventually develop recurrent disease that is resistant to tamoxifen treatment. There are many proposed mechanisms of tamoxifen resistance, including, but not limited to, ligand-independent activation of the estrogen receptor [1] and tamoxifen acting as an agonist via crosstalk with other transcription factors [5]. Both of these resistance mechanisms have been connected to the activity of extracellular signal regulated kinases (ERK), one of the Mitogen-Activated Protein Kinases (MAPKs) [1, 5]. The MAPK family consists of three branches: ERK, JNK and p38. The activity of these kinases is stimulated by growth signals (ERK, JNK, and p38), cytokines (JNK, p38) and cellular stress (JNK, p38) [6, 7] and they are activated by dual threonine-tyrosine phosphorylation on residues present in a TXY motif in their activation loop [8]. ERK is known to contribute to ligand-independent activation of ERalpha through phosphorylation of Ser118 [9]. ERK activity has also been associated with endocrine therapy resistance and decreased survival of breast cancer patients [4]. Pharmacological inhibition of ERK activity *in vitro* has been shown to reverse the tamoxifen resistant phenotype in breast [10] and several other cancer types [11, 12].

Endogenously, ERK activation is inhibited through the activity of Mitogen-activated protein kinase phosphatases (MKPs). The MKPs are a family of eleven dual-specificity phosphatases that attenuate MAP Kinase activity through dephosphorylation of threonine and tyrosine residues present in the TXY motif [6, 7]. Although MKPs have been associated with a variety of cancer types, upregulation of MKP-1 and MKP-2 expression has been reported in breast cancer [13]. MKP-1 and MKP-2 are nuclear phosphatases that dephosphorylate ERK, JNK and p38 [14-16]. Their transcription can be induced by ERK phosphorylation of transcription factors [17]. Although the role of the MAPK signaling pathway in breast cancer development, progression and tamoxifen resistance is well documented [9, 18], very little is known about the role of MKPs in tamoxifen response and sensitivity. Here, we characterize MKP expression in breast cell lines and show that MKP-2 levels increase following tamoxifen treatment, whereas MKP-1 expression is unaffected. Overexpression of MKP-2 results in decreased estrogen-induced cell proliferation and increased sensitivity to tamoxifen, potentially by abrogation of ERK phosphorylation.

## RESULTS AND DISCUSSION

### Characterization of MKP expression in breast cell lines

**Figure 1:**
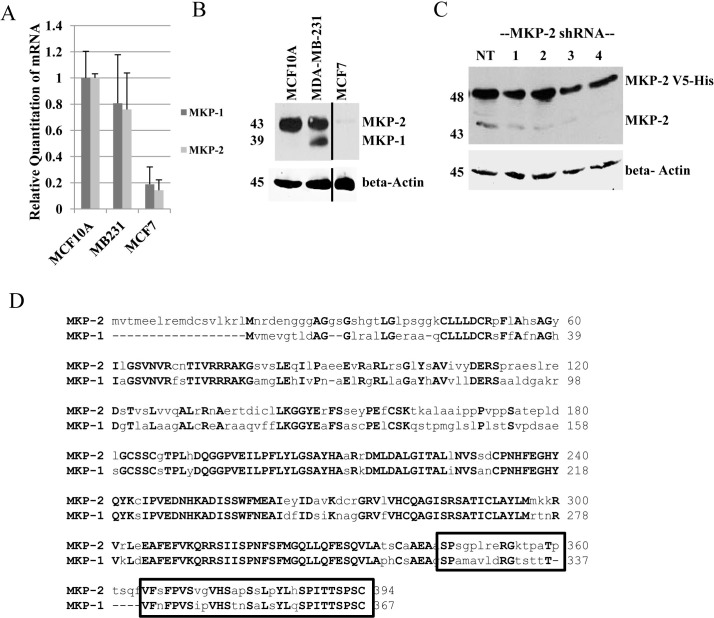
Characterization of MKP-1 and MKP-2 in breast cell lines A- Untreated whole cell lysates of MCF10A, MDA-MB-231, and MCF7 cells were analyzed by western blot and probed with anti-MKP-1 antibody. Actin was used as a loading control. B. Real time PCR using *MKP-1*- and *MKP-2*-specific primers were performed to assess mRNA expression in MCF10A, MDA-MB-231 and MCF7 cells. GAPDH was used as an internal control. C- MCF7-MKP-2 cells were transiently transfected with either the non-silencing control (NT) or one of four MKP-2 shRNA constructs. Whole cell lysates were subjected to western blot analysis with anti-MKP-1 antibody. β-Actin was used as a loading control. D- MKP-1 [GenBank:NP_004408.1] and MKP-2 [GenBank:NP 001385.1] amino acid sequences were aligned using ClustalW2 software. Boxed region represents the location of the MKP-1 antibody epitope. Uppercase bold letters represent identical amino acid residues.

To study the regulation of MKPs and their effect on MAPK signaling in tamoxifen sensitivity, we identified a cell line model suitable for expression of exogenous MKP proteins. A panel of three breast cell lines (non-tumorigenic MCF10A, ER-negative MDA-MB-231 and estrogen receptor positive MCF7) was screened for MKP expression using real-time RT-PCR and western blot analysis. Real-time PCR analysis showed that MCF10A and MDA-MB-231 cells express similar levels of both *MKP-1* and *MKP-2* mRNA, whereas MCF7 cells expressed low levels of both *MKP-1* and *MKP-2* mRNAs (Figure 1A). Western blot analysis with anti-MKP-1 antibody showed the presence of MKP-1 (39 kDa) and a 43 kDa protein in MDA-MB-231 cells, whereas only the 43 kDa band was seen in MCF10A cells. Trace amounts of the 43 kDa protein were detected in MCF7 cells (Figure 1B). The estrogen-receptor positive T47D cell line showed results similar to MCF7 cells (data not shown). To confirm the identity of the 43 kDa band detected by the anti-MKP-1 antibody, MCF7 cells engineered to overexpress a V5-His tagged MKP-2 (48 kDa) were transiently transfected with either a non-silencing control shRNA or one of four MKP-2 shRNA constructs. MKP-2 shRNAs reduced both the exogenous 48 kDa and endogenous 43 kDa bands compared to the non-silencing controls, suggesting that the 43 kDa band detected by the MKP-1 antibody is MKP-2 (Figure 1C). ClustalW alignment of MKP-1 and MKP-2 amino acid sequences revealed that 29 of the 50 amino acids known to contain the MKP-1 antibody epitope are identical (Figure 1D). These data may explain the recognition of MKP-2 by the MKP-1 antibody. MCF7 was chosen as the model cell line for further studies due to the weak and undetectable levels of MKP-2 and MKP-1, respectively.

### MKP-2, but not MKP-1 expression increases following tamoxifen treatment

**Figure 2:**
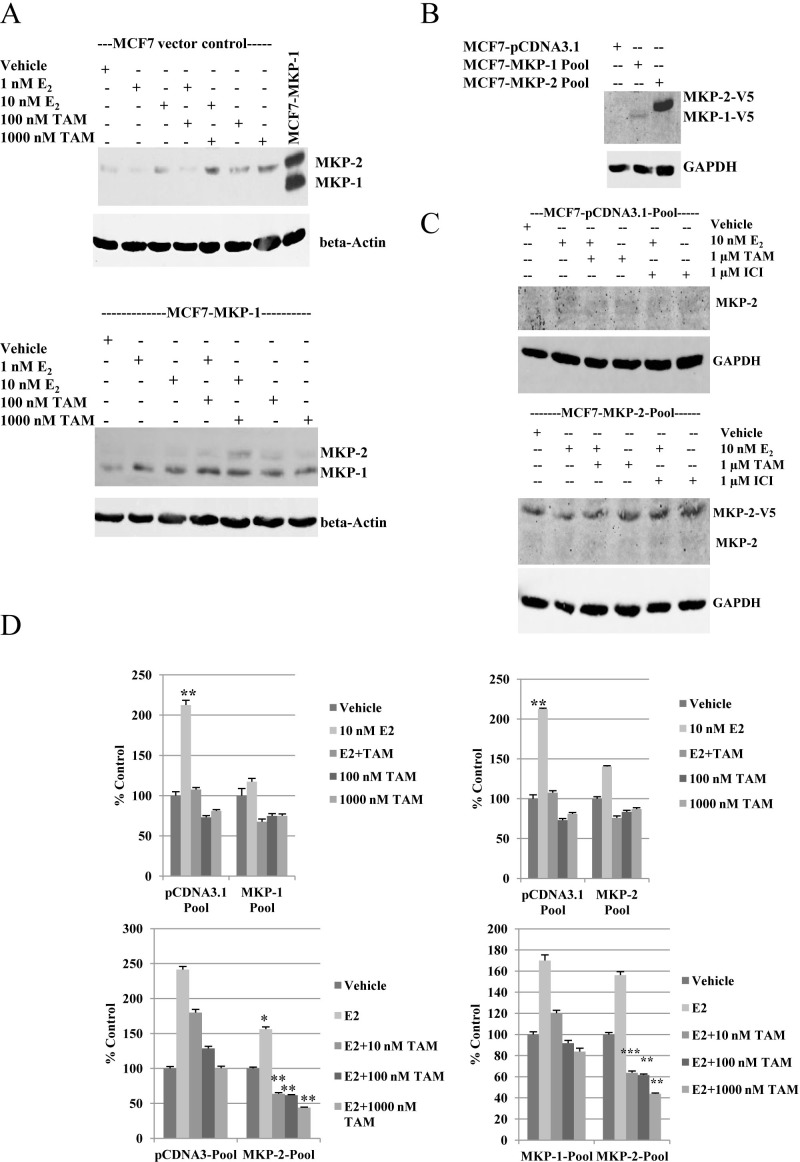
Overexpression of MKP-1 or MKP-2 inhibits estrogen-induced cell proliferation, but retains tamoxifen sensitivity A- Western blot analysis of MKP-1 and MKP-2 in MCF7 vector control and MCF7-MKP-1 clones following hormonal treatments. Membranes were probed with anti-MKP-1 antibody. MCF7-MKP-1 cells were used as a positive control to locate the MKP-1 band. β-Actin was used as a loading control. B- Western blot analysis of V5-tagged MKP-1 and MKP-2 in pooled populations of MCF7-pcDNA3.1 vector control, MCF7-MKP-1 and MCF7-MKP-2 cells. Membrane was probed with anti-V5 antibody and GAPDH was used as a loading control. C- Western blot analysis of MKP-2 in MCF7-pcDNA3.1 and MCF7-MKP-2 pools following hormonal treatments. Membranes were probed with anti-MKP-1 antibody. D- Regulation of growth by hormones in MCF7-pcDNA3.1, MCF7-MKP-1 and MCF7-MKP-2 pools. MCF7-pCDNA3.1-Pool cells and MCF7-MKP-2-Pool cells were treated with vehicle, 10 nM E_2_, E_2_+TAM, 100 nM TAM or 1000 nM TAM and MTT analysis was performed. Cells were treated on days 1 and 4. Absorbance was read on day 7. Results are representative of at least three independent experiments with samples plated in triplicate. Bottom panels- MCF7-pCDNA3.1-Pool cells, MCF7-MKP-1-Pool cells and MCF7-MKP-2-Pool cells were treated with vehicle, E_2_ or E_2_ plus one of three increasing concentrations of TAM and and MTT assay was performed. Cells were treated on days 1 and 4 and absorbance was read on day 7. Results are representative of two independent experiments with samples plated in triplicate. Statistical analysis was done using Student's t-test. * = p<0.05, ** = p <0.01, *** = p<0.001

To determine the effect of tamoxifen on MKP-1 protein expression, single clones of cells expressing empty vector or MKP-1 (MCF7-MKP-1) were cultured in phenol-red free medium supplemented with charcoal-stripped FBS overnight and then treated for twenty-four hours with either 1 or 10 nM E_2_, 100 nM or 1000 nM TAM or the combination of E_2_ and 100-fold molar excess TAM. Western blot analysis of whole cell lysates showed no detectable changes in levels of exogenous MKP-1 protein expression and endogenous MKP-1 remained undetectable (Figure 2A). However, treatment with TAM increased MKP-2 protein levels in both MCF7 vector control and MCF7-MKP-1 cells (Figure 2A).

### MKP-1 and MKP-2 overexpression do not alter tamoxifen sensitivity

To better reflect the heterogeneity present in a tumor, stable clones of MCF7 cells transfected with empty pCDNA3.1, pCDNA3.1-MKP-1 or pCDNA3.1-MKP-2 were pooled. Expression of exogenous MKP-1 and MKP-2 were verified by western blot with anti-V5 antibody. Bands corresponding to 44 and 48 kDa, representative of V5-tagged MKP-1 and MKP-2, respectively, were detected (Figure 2B). Whole cell lysates of MCF7-pCDNA3.1-Pool and MCF7-MKP-2-Pool cells were analyzed by western blot following treatment with E_2_, TAM, ICI or the combination of E_2_+TAM or E_2_+ICI (Figure 2C). Low levels of endogenous MKP-2 protein were detected following TAM treatment in both MCF7-pCDNA3.1-Pool and MCF7-MKP-2-Pool cells.

To assess the effect of MKP-1 overexpression on MCF7 cell proliferation in response to estrogen or tamoxifen treatment, MCF7-MKP-1-Pool cells were treated with E_2_, TAM or a combination of E_2_ and 100-fold molar excess TAM. Compared to vector control cells that responded to E_2_ treatment with a two-fold increase in cell proliferation, MCF7-MKP-1-Pool cell proliferation was unaffected by E_2_. However, MCF7-MKP-1-Pool cells exhibited similar levels of TAM sensitivity as vector control cells (Figure 2D). Taken together, these results suggest that MKP-1 overexpression does not contribute to alterations in TAM sensitivity but diminishes stimulation by estrogen. Analyses of vector control and MKP-2 overexpressing MCF7 cells showed similar data as MCF7-MKP-1-Pool cells. MCF7-MKP-2-Pool cells remained sensitive to TAM treatment (Figure 2D). To verify these data, growth responses to E_2_ in the presence of increasing concentrations of TAM were determined. Vector control, MKP-1- and MKP-2-overexpressing cells were treated with E_2_ alone or a combination of E_2_ and 1-, 10- or 100-fold molar excess TAM. Overexpression of MKP-2 caused a significant (p<0.05) suppression of the E_2_-induced cell proliferation observed in vector control (Figure 2D). Treatment with a combination of E_2_ with 1, 10 or 100-fold molar excess of TAM resulted in dose dependent inhibition of MCF7-MKP-1-Pool and MCF7-MKP-2-Pool cell proliferation similar to vector control cells. However, compared to vector control and MKP-1 overexpressing cells, MKP-2 overexpression conferred greater TAM sensitivity, as significant (p<0.01) decreases in cell proliferation were observed at all three doses of TAM.

### Overexpression of MKP-1 and MKP-2 abrogates ERK activity in MCF7 cells

**Figure 3:**
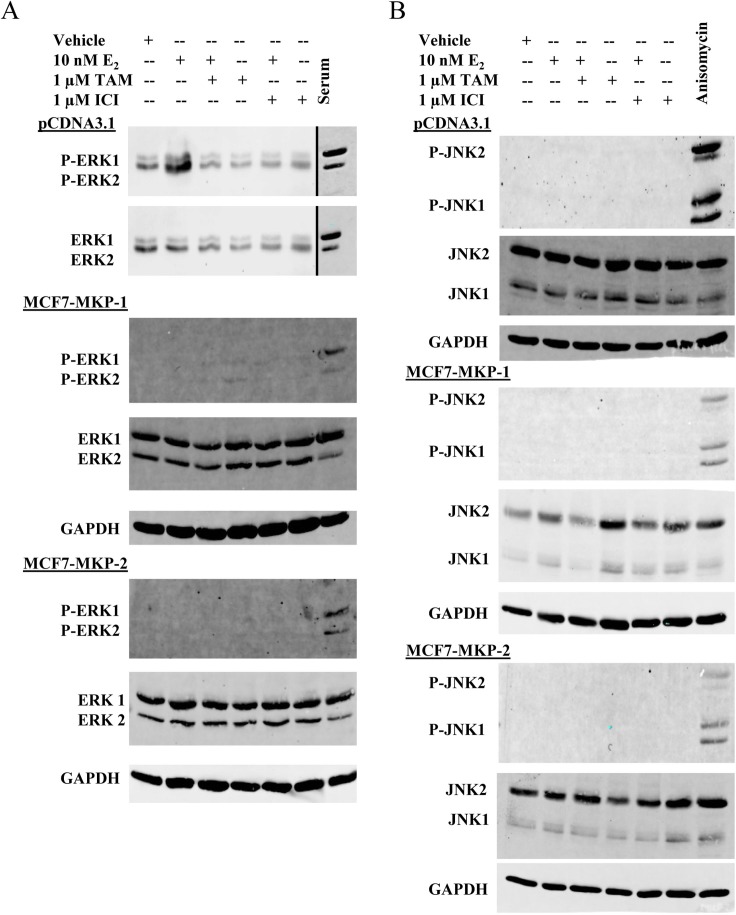
Overexpression of MKP-1 or MKP-2 abrogates ERK activity in MCF7 cells A- Whole cell lysates of MCF7-pCDNA3.1-Pool, MCF7-MKP-1-Pool and MCF7-MKP-2-Pool cells were subjected to western blot analysis of phospho-ERK1/2 and total ERK1/2. Lysates prepared from MCF7 cells stimulated with serum following serum starvation were used as a positive control for ERK1/2 phosphorylation. GAPDH was used as a loading control. B- Western blot analysis of phospho-JNK1/2 and total JNK1/2. Lysates prepared from MCF7 cells treated with 10 ng/mL anisomycin were used as a positive control for JNK phosphorylation. GAPDH was used as a loading control.

The decrease in proliferation of MCF7-MKP-1-Pool or MCF7-MKP-2-Pool cells in response to E_2_ treatment, as well as the increase in MKP-2 expression following TAM treatment (Figure 2A) suggests that these MKPs impact the activities of MAPKs that drive the proliferation of these cells. To determine which of the MAPKs are dephosphorylated by MKP-1 and MKP-2, western blot analysis of ERK1/2 and JNK1/2 (two MAPK family members associated with cell proliferation) was performed. Vector control, MCF7-MKP-1-Pool and MCF7-MKP-2-Pool cells were depleted of E_2_ and then treated with E_2_, TAM, ICI or combinations of E_2_+TAM or E_2_+ICI. Phospho-ERK1/2 levels were increased in response to E_2_, but returned to basal levels upon the addition of TAM (Figure 3A). Overexpression of MKP-1 or MKP-2 completely abolished ERK1/2 activation regardless of treatment condition (Figure 3A). These results confirm the biological activity of the exogenously expressed MKP-1 and MKP-2 and suggest that the decreases in E_2_-induced proliferation of MKP-1 and MKP-2 overexpressing cells compared to vector control cells are likely due to ERK1/2 dephosphorylation. JNK1/2 activation was not detectable in vector control, MCF7-MKP-1-Pool or MCF7-MKP-2-Pool cells. These data further suggest that TAM-induced cell death of these cells is not dependent on JNK signaling, but rather that ERK is the major MAPK driving their proliferation.

### MKP-2 expression is increased in tamoxifen resistant MCF7 cells

**Figure 4:**
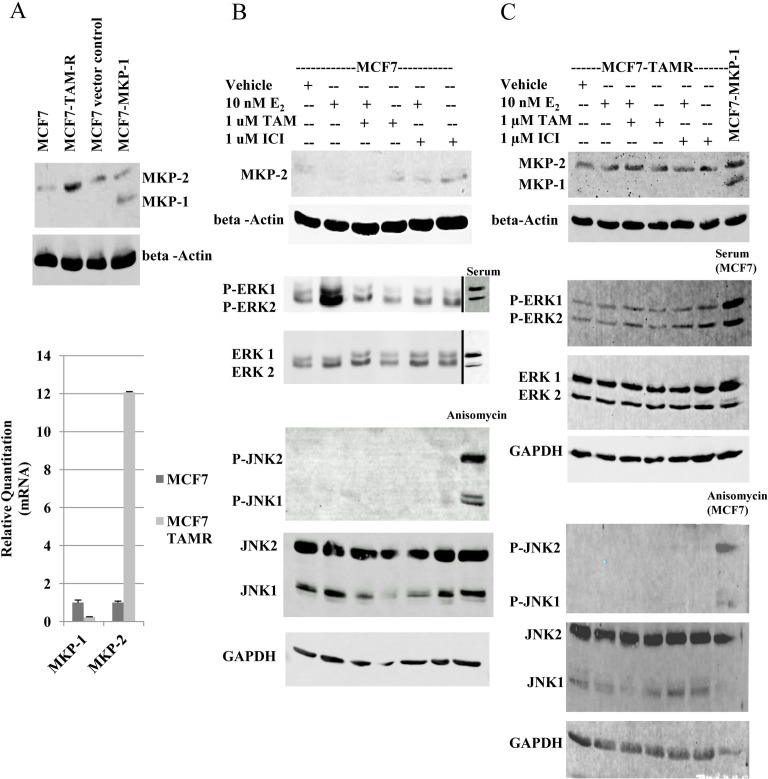
MKP-2 expression and activity analysis in MCF7 and MCF7-TAMR cells A. Top panel. Western blot analysis of MKP-1 and MKP-2 in MCF7 and MCF7-TAMR cells. Positions of MKP-1 and MKP-2 were located with MCF7-pEGFP and MCF7-MKP-1 cell lysates. Membranes were probed with anti-MKP-1 and beta-actin. Lower panel. Real-time RT-PCR analysis of MKP-1 and MKP-2 mRNAs in MCF7 and MCF7-TAMR cells. MKP levels are expressed relative to GAPDH. B and C- Western blot analysis of MKP-1, MKP-2, ERK1/2 and JNK1/2 in MCF7 (B) and MCF7-TAMR (C) cells following hormonal treatments. Whole cell lysates prepared from MCF7 cells stimulated with serum following serum deprivation were used as the positive control for ERK1/2 phosphorylation and MCF7 cells treated with 10 ng/ml anisomycin served as positive control for JNK phosphorylation. GAPDH was used as a loading control.

Since the data obtained thus far were with cells engineered to constitutively express MKP-1 or MKP-2, we verified the roles of these MKPs under physiological conditions using an isogenic model of acquired tamoxifen resistance (MCF7 and MCF7-TAMR cells). Compared to MCF7 cells, a 12-fold increase in MKP-2 mRNA expression was observed in MCF7-TAM-R cells, whereas MKP-1 expression was negligible as in MCF7 parental cells (Figure 4A). Western blot analysis verified real-time RT-PCR data, as a 3.5-fold increase in the level of MKP-2 was detected in MCF7-TAMR cells (Figure 4A).

Whole cell lysates of parental MCF7 cells treated with E_2_, TAM, ICI or the combination of E_2_+TAM or E_2_+ICI were analyzed by western blot for MKP-1, MKP-2, ERK1/2 and JNK1/2 levels (Figure 4B). Low levels of MKP-2 protein were detectable following treatment with anti-estrogens in MCF7 cells. ERK1/2 activation was detected following E_2_ treatment and returned to basal levels upon the addition of TAM. JNK activation was not detected. Analysis of MKP-2 regulation in MCF7-TAMR cells following treatment with E_2_, TAM, ICI or combinations of E_2_+TAM or E_2_+ICI showed that MKP-2 levels were unaffected by treatments and MKP-1 expression remained undetectable (Figure 4C). Consistent with reported data [19], ERK1/2 remained active regardless of treatment condition (Figure 4C). As with MKP-1 or MKP-2 overexpressing cells (Figure 3), JNK activation was not detected in the MCF7-TAMR cells (Figure 4C). These data corroborate the data from Figures 2 and 3 and suggest that MKP-2 expression is upregulated by tamoxifen treatment.

## CONCLUSIONS

**Figure 5:**
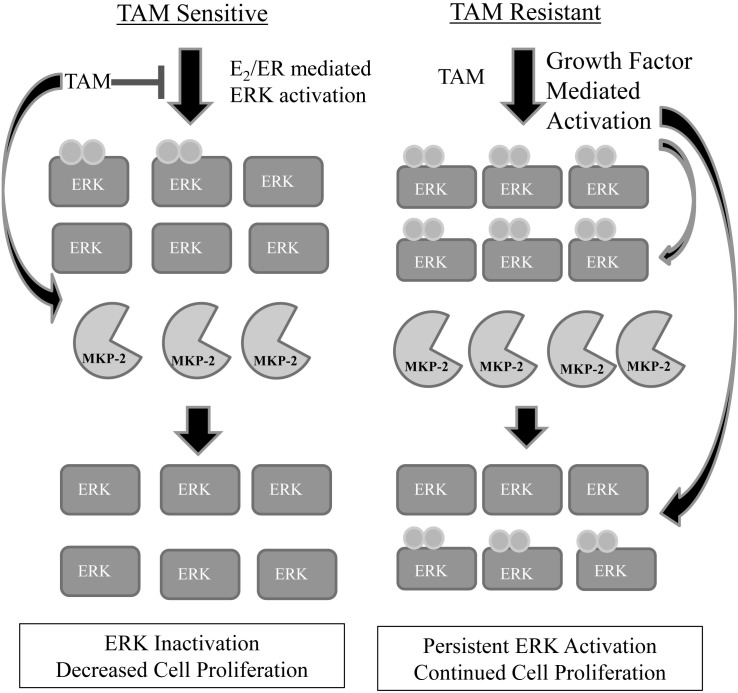
Proposed model of MKP-2 regulation in tamoxifen sensitive vs. tamoxifen resistant cells In tamoxifen sensitive cells, low levels of phosphorylated ERK1/2 are present, indicating that cell-growth signaling pathways are activated. Following treatment with TAM, MKP-2 protein expression is increased with resultant dephosphorylation of ERK1/2 and slowing or elimination of cell proliferation. In tamoxifen resistant cells, phosphorylated ERK1/2 is constitutively present at higher levels than in tamoxifen sensitive cells. MKP-2 protein expression is upregulated in an attempt to return phospho-ERK1/2 levels to that of a tamoxifen sensitive cell. The levels of active ERK may be too high for MKP-2 to completely dephosphorylate ERK1/2, resulting in continued cell survival. Additionally, previous work in our lab has shown that active ERK is able to phosphorylate MKP-2, leading to its stabilization, which might be an additional factor in the higher protein levels seen in tamoxifen resistant cells compared to tamoxifen sensitive cells.

MAP Kinase Phosphatases are key components of MAPK signaling and regulation. Previous studies have reported a link between MKPs and therapy resistance in breast and a variety of other cancers [7, 20]. However, the role of MKPs in tamoxifen response has not been studied. We show here that treatment with TAM stimulates the expression of MKP-2, but does not have the same effect on MKP-1. Similar analysis of MCF7-TAMR cells showed constitutive expression of MKP-2, but no detectable levels of MKP-1. Overexpressing MKP-1 or MKP-2 decreased the ability of MCF7 cells to proliferate in the presence of E_2_, TAM or their combination due to abrogation of ERK1/2 activation rather than JNK1/2 activation. These results also indicate that when expressed above physiological levels, the substrate specificity of MKP-1 (ERK<<JNK, p38) is lost. MCF7-TAMR cells, which have high levels of activated ERK, exhibited a constitutive level of MKP-2 expression. Taken together, these results suggest that MKP-2 mediated ERK inactivation sensitizes breast cancer cells to TAM treatment. We posit that MKP-2 expression is upregulated in TAM resistant cells to potentially help return cells to a TAM sensitive state. In our proposed model (Figure 5), in TAM sensitive cells, which are dependent on E_2_ signaling for survival, MKP-2 is upregulated following TAM treatment to inactivate ERK, which results in slowing of cell proliferation and subsequent cell death. In TAM resistant cells, which are not sensitive to E_2_, ERK activation is present at higher levels than in TAM sensitive cells. This activation is mediated by increased growth factor signaling, which is known to occur in TAM resistant tumors [21, 22]. Although these cells show higher levels of MKP-2 gene expression, the levels of phospho-ERK1/2 in these cells are probably too high for MKP-2 to completely eliminate its activity. Previous work in our lab has shown that activated ERK can stabilize MKP-2 by phosphorylating MKP-2 on Ser386 and Ser391 [23]. Since ERK activity is higher in TAM resistant cells, its ability to potentially modify MKP-2 post-translationally might contribute to the higher levels of MKP-2 protein in these cells. Thus, despite the constant presence of MKP-2 in TAM resistant cells, it is unable to halt ERK-mediated proliferation rendering the drug ineffective.

The relationship between cell signaling pathways and the endogenous molecules that attenuate their activity is important and often understudied in the context of cancer treatment. While increasing the expression/activity of MKP-2 to sensitize cells to tamoxifen therapy is not a viable therapeutic strategy at this time, our results support the importance of continuing to develop clinically effective ways to reduce ERK activity in breast cancer cells. The idea that MKP-2 plays a role in chemotherapy response is also supported by a recent study by Balko *et al* [24], in which molecular profiling of basal-like breast cancer tissues revealed that loss of MKP-2 expression was associated with increased ERK pathway activation and reduced disease-free survival following neo-adjuvant chemotherapy. The authors also suggested that MKP-2 expression could be used as a biomarker for MEK inhibitor sensitivity in these patients [24]. In light of these results, MKP-2 might also be useful as a marker for sensitivity in tumors that are candidates for TAM treatment. However, further development of antibodies and reagents that can selectively differentiate the expression and activities of MKP-2 from MKP-1 are necessary for this strategy to be effective. The connection between TAM treatment, MKP-2 activation and inhibition of ERK activity in these cells also needs to be investigated further to determine how best to translate these findings into clinical benefit.

## MATERIALS AND METHODS

### Cell Lines and Culture Conditions

MDA-MB-231 cells were cultured in Dulbecco's Minimal Essential Medium (DMEM) supplemented with 10% fetal bovine serum (FBS)[25]. MCF10A cells were grown in DMEM/F12 (1:1) medium supplemented with 5% FBS, 10 μg/mL insulin, 20 ng/mL epidermal growth factor, 100 ng/mL cholera enterotoxin and 0.5 μg/mL hydrocortisone [25]. MCF7 cells were maintained in DMEM/F12 (1:1) medium supplemented with 5% FBS, 10 μg/mL insulin and 0.5 nM 17-beta-estradiol [26]. Tamoxifen resistant MCF7 cells (MCF7-TAMR) were generated by gradual exposure to increasing concentrations of 4-hydroxy-tamoxifen (TAM) over a period of six months. MCF7-TAMR cells are able to tolerate exposure to 10 μM TAM. MCF7-TAMR cells were routinely maintained in DMEM/F12 medium supplemented with 5% FBS, 10 μg/mL insulin and 1 μM TAM (Gerard and Shekhar, manuscript in preparation). MCF7 cells stably expressing empty vector (pEGFP or pCDNA3.1), MKP-1 [27] or MKP-2 [23] were maintained in the same medium as MCF7 cells and stable clones were selected with 500 μg/mL G418. All cell lines were maintained in a 37°C incubator with a humidified atmosphere consisting of 5% CO_2_. For experiments involving treatment with 17-beta-estradiol (E_2_), tamoxifen (TAM) or ICI 182,780 (ICI), cells were depleted of endogenous hormones by culturing in phenol red free media supplemented with charcoal-stripped FBS and 10 μg/mL insulin.

### Generation of MKP-1 and MKP-2 Overexpressing Cell Lines

Pooled populations of MCF7 cells overexpressing full-length human MKP-1, MKP-2, or the empty pCDNA3.1 expression vector were generated by stable transfection using Metafectine Easy transfection reagent (Biontex). Stable clones expressing V5-His tagged MKP-1 or MKP-2 were selected with G418 antibiotic (500 μg/mL) and pooled to minimize clonal bias. Stable pools of MCF7 cells expressing empty vector, MKP-1 or MKP-2 were maintained in DMEM/F12 (1:1) medium supplemented with 5% FBS, 10 μg/mL insulin, 0.5 nM E_2_ and 500 μg/mL G418.

### Reagents

17-beta-estradiol, G418 and 4-OH-tamoxifen were purchased from Sigma. ICI 182,170 was purchased from Tocris. Anisomycin (Sigma) was a gift from Dr. Raymond Mattingly. Human recombinant insulin was purchased from Gibco.

### Antibodies

MKP-1 (C-19) antibody was purchased from Santa Cruz Biotechnology. PhosphoPlus SAPK/JNK (Thr183/Tyr185) Antibody Kit, PhosphoPlus p44/p42 MAPK (ERK1/2) (Thr202/Tyr204) Antibody Kit, GAPDH antibody, goat-anti-mouse-HRP and goat-anti-rabbit-HRP secondary antibodies were purchased from Cell Signaling Technology. Anti-V5 antibody was purchased from Invitrogen. Anti-beta-actin antibody was purchased from Sigma Aldrich. When using the Odyssey scanner, Alexa-Fluor 680 conjugated to goat-anti-rabbit or goat-anti-mouse (Invitrogen) or IRDye 800 conjugated to goat-anti-rabbit or goat-anti-mouse (Li-Cor) were used as secondary antibodies.

### Western Blot Analysis

Whole cell lysates were prepared as described previously [28] and aliquots of lysates containing 30 μg protein were subjected to SDS-PAGE and western blot analysis of MKP-1, MKP-2, phospho-ERK1/2, total ERK1/2, phospho-JNK1/2, JNK1/2, actin and GAPDH. Proteins were detected either with the Amersham ECL Plus Reagent(GE Healthcare) or by Odyssey Infrared imaging.

### RNA Isolation, cDNA Synthesis and Real-Time RT-PCR Analysis

Total RNA was isolated using TRIzol Reagent (Life Technologies) and cDNAs were synthesized using the SuperScript III Reverse Transcriptase kit (Invitrogen) using 5 μg total RNA and random primers. cDNA quality and specificity of the gene-specific primers were verified by semi-quantitative PCR using gene specific primers. RT-PCR was carried out using the Choice-Taq DNA polymerase (Denville) and the following conditions: 95°C for 2 minutes, followed by thirty-five cycles of 95°C for 15 seconds, 55°C for 30 seconds and 72°C for 60 seconds and a final 5 minute extension step at 72°C. The following primer sequences were used for semi-quantitative and Real Time PCR: GAPDH forward (5'-ATC AAG AAG GTG GTG AAG CAG-3', +946 to 966, NM_002046.4), GAPDH reverse (5'-TGT CGC TGT TGA AGT CAG AGG-3', +1042 to 1022; NM_002046.4), MKP-1 forward (5'-GAA GTG GGC ACC CTG GAC GC-3', +258 to 277; NM_004417.3), MKP-1 reverse (5'-TGG CCG GCG TTG AAA GCG AA-3', +364 to 345; NM_00417.3), MKP-2 forward (5'-GAG TCC GCG GTC CTC TCT CGT-3', +494 to 515; NM_001394.6) and MKP-2 reverse (5'-CCT CGC GGT CAC ATA GCA GTC G-3', +642 to 623; NM_001394.6). Real-Time RT-PCR was performed using the SYBR Green PCR core reagent kit (Applied Biosystems) on the Step One Plus Real-Time PCR System. Thermal cycling conditions were: 95°C for 10 minutes followed by forty cycles of 95°C for 15 seconds and 60°C for 1 minute. Relative levels were determined using the ∆∆Ct method and GAPDH used as the internal control. Each sample was run in duplicate and and results presented are representative of at least three independent experiments.

### shRNA Suppression of MKP-2

Four *MKP-2* shRNAs subcloned into the pGIPZ vector (Open Biosystems) were tested. The *MKP-2* targeting sequences were as follows: shRNA 1(RHS4430-101067857): CCCCAGTGGAAGATAACCACAA; shRNA 2 (RHS4430-101069298): ATTCGGTCAACGTGCGCTGTAA; shRNA 3 (RHS4430-101073316): ACTGGTTCATGGAAGCCATAGA; shRNA 4 (RHS4430-98713911): AGCCTACCTGATGATGAAGAAA. Constructs were transiently transfected into MCF7-MKP-2 cells using Lipofectamine 2000 according to the manufacturer's protocol. Non-silencing shRNA was used as a negative control.

### MTT Assay

MCF7-pCDNA3.1-Pool, MCF7-MKP-1-Pool or MCF7-MKP-2-Pool cells (1x10^3^ cells per well) were seeded in 96 well plates (day 0) and treated with vehicle (EtOH, 0.1% v/v), 10 nM E_2_, 100 or 1000 nM TAM, or a combination of 10 nM E_2_ and 1-, 10- or 100-fold molar excess TAM. Treatments were refreshed on day 4. Assays were terminated on day 7 or when control wells reached 85% confluency, whichever was longer. Cell viability was assessed by MTT assay (Sigma) and measured on a Synergy 2 plate reader (BioTek) with Gen5 1.10 software.

### Statistical Analysis

Statistical analysis was done using Student's t-test. p<0.05 was considered significant.

### Competing Interests

The authors have no competing interests to declare.
