# Erratum: Functional analysis of MKP-1 and MKP-2 in breast cancer tamoxifen sensitivity

**DOI:** 10.18632/oncotarget.26258

**Published:** 2018-10-16

**Authors:** Kelly K. Haagenson, Jessica Wei Zhang, Zhengfan Xu, Malathy P.V. Shekhar, Gen Sheng Wu

**Affiliations:** ^1^ Department of Oncology, Wayne State University School of Medicine, Detroit, MI; ^2^ Barbara Ann Karmanos Cancer Institute, Detroit, MI; ^3^ Department of Pathology, Wayne State University School of Medicine, Detroit, MI

**This article has been corrected:** During production, the ending page number for this article was listed incorrectly. The page count has now been adjusted to show the proper pagination.

Original article: Oncotarget. 2014; 5:1101-1110. https://doi.org/10.18632/oncotarget.1795

